# The complete chloroplast genome of *Semenovia thomsonii* (Tordylieae: Apiaceae), a new record from Xizang, China

**DOI:** 10.1080/23802359.2021.1935340

**Published:** 2021-06-15

**Authors:** Qun-Ying Xiao, Tu Feng, Qiang Luo, Xing-Jin He

**Affiliations:** a School of Ecological Engineering, Guizhou University of Engineering Science, Bijie, PR China; b Key Laboratory of Bio-Resources and EcoEnvironment of Ministry of Education, College of Life Sciences, Sichuan University, Chengdu, PR China

**Keywords:** *Semenovia thomsonii*, complete chloroplast genome, phylogenetic analysis

## Abstract

*Semenovia thomsonii* is a perennial herb native to India. In this study, we assembled and annotated the complete chloroplast (cp) genome of a specimen collected from Xizang, China, using whole genome next-generation sequencing. The cp genome is circular in structure and 147,137 bp in length, consisting of one large single-copy (LSC) region of 92,885 bp, one small single-copy (SSC) region of 17,448 bp, and a pair of inverted repeat regions of 36,804 bp. The overall GC content of the genome is 37.6%. The cp genome was predicted to contain 129 genes, including 85 protein-coding, 36 *tRNA*, and eight *rRNA*. Phylogenetic analysis of *S. thomsonii* and 21 cp genomes in the Apiaceae fully resolved *S. thomsonii* in a clade with *S. gyirongensis*, and *S. transiliensis*. These genetic data represent the first confirmed report of *S. thomsonii* from Xizang, China and provide useful information to the phylogenetic history of the genus *Semenovia*.

*Semenovia thomsonii* (C. B. Clarke) Manden (Apiaceae, Apioideae), is naturally distributed in Jammu, Kashmir, Himachal Pradesh and occurs throughout India (Mukherjee and Constance 1993). Examination of specimens from Zhada County, Xizang, we identified *S. thomsoni* from the collection (8180, KUN; 76-8180, PE; 76-9163, QTPMB). The specimens were misidentified as *Heracleum millefolium* Diels. In August 2015, we successfully collected the same samples according to the collection records and further confirmed based on morphology, the occurrence of *S. thomsonii* in China. In the most updated checklist of the Chinese Umbelliferae, *S. thomsonii* is recognized in China (Pimenov 2017). To confirm the presence of *S. thomsonii* in China, we performed next-generation sequencing on a specimen from Xizang and compared its genome to previously published species of *Semenovia*.

The mature leaves of *S. thomsonii* were collected from a rocky slope near Seerdi village (32°11′58.02″N, 79°10′58.68″E, altitude 4200 m), Qusong country, Zhada County, Xizang, China and preserved them using silica gel for future study. A voucher specimen (voucher number: xqy2015081901) was deposited in the herbarium of the Natural History Museum of Sichuan University (SZ). Herbarium acronyms followed Thiers (2016). Total genomic DNA of *S. thomsonii* was isolated using the Plant Genomic DNA Kit (TIANGEN Biotech., Beijing, China) and sequenced on an Illumina HiSeq × Ten platform (Illumina, San Diego, CA). Approximately, 5 Gb of raw data were generated through pair-end 150 bp sequencing. Adapters and low-quality reads were removed and high-quality reads were used for the cp genome assembly using SOAPdenovo2 (Luo et al. 2012). The resulting contigs were linked based on overlapping regions after being aligned to *S. gyirongensis* Q.Y. Xiao & X.J. He (NC_042912) using Geneious version 11.0.4 (Kearse et al. 2012). The complete chloroplast (cp) genome of *S. thomsonii* was annotated in Geneious and submitted to GenBank (accession number: MW371294). The genome annotation was performed by aligning with the cp genomes of related species.

The cp genome of *S. thomsonii* exhibited a general quadripartite structure typical of higher plants. The cp genome is 147,137 bp in length and contains a large single-copy region (LSC) of 92,885 bp and a small single-copy region (SSC) of 17,448 bp, separated by two identical inverted repeat regions (IRa and IRb, 18,402 bp). The overall GC content was 37.6% and the plastome contained 129 genes, including 85 protein-coding, eight rRNA, and 36 tRNA.

To confirm the phylogenetic position of *S. thomsonii* within the family of Apiaceae, a total of 21 complete cp genomes of Apiaceae were obtained from GenBank, designating *Bupleurum boissieuanum* and *B. falcatum* as out-groups. The 22 complete cp sequences were aligned using MAFFT version 7 (Katoh and Standley 2013) and maximum likelihood (ML) analysis was conducted using RAxML (Stamatakis 2014) with 1000 bootstraps under the GTRGAMMAI substitution model. The phylogenetic tree (Figure 1) indicated that *S. thomsonii* was closely related to *S. gyirongensis* and *S. transiliensis* Regel & Herder. These results are similar to those found by Logacheva et al. (2010) and Xiao et al. (2018). This analysis represents the first genetic confirmation of *S. thomsonii* in China and the first published cp genome. The data will provide useful information for phylogenetic studies and conservation genetics in the Apiaceae.

**Figure 1.**
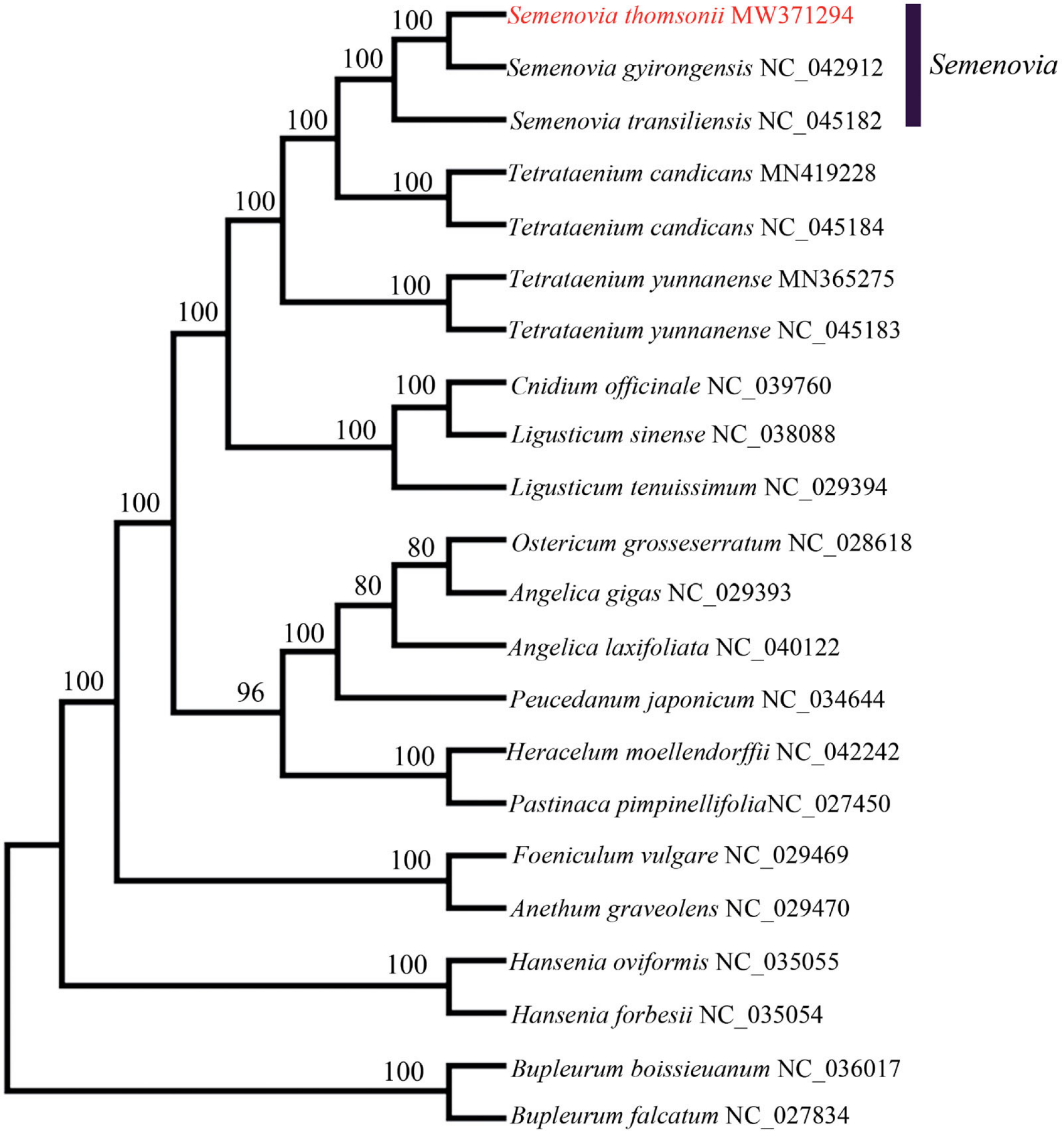
Phylogenetic analysis of *S. thomsonii* and related Apiaceae based on complete chloroplast genome sequences. Bootstrap values based on 1000 replicates a listed at the nodes and GenBank accession follow the binomials.

## Research involving human participants and/or animals

This article does not contain any studies with human participants or animals performed by any of the authors.

## Data Availability

Chloroplast data supporting this study are openly available in GenBank at nucleotide database, https://www.ncbi.nlm.nih.gov/nuccore/MW371294. Associated BioProject, https://www.ncbi.nlm.nih.gov/bioproject/PRJNA730370, BioSample accession number at https://www.ncbi.nlm.nih.gov/biosample/SAMN19229653 and Sequence Read Archive at https://www.ncbi.nlm.nih.gov/sra/SRR14561442.
